# Risk stratification and real-world management of pediatric AAORCA: discordance between anatomical features and clinical symptoms

**DOI:** 10.3389/fcvm.2026.1876090

**Published:** 2026-07-15

**Authors:** Jinjin Yu, Shushui Wang, Qinchang Chen, Ping Zhu, Wei Pan

**Affiliations:** 1 Department of Cardiac Surgery, Guangdong Cardiovascular Institute, Guangdong Provincial People’s Hospital, Guangdong Academy of Medical Sciences, Guangzhou, Guangdong, China; 2 Department of Pediatric Cardiology, Guangdong Cardiovascular Institute, Guangdong Provincial People’s Hospital, Guangdong Academy of Medical Sciences, Guangzhou, Guangdong, China; 3 Department of Maternal-Fetal Cardiology, Guangdong Cardiovascular Institute, Guangdong Provincial People’s Hospital, Guangdong Academy of Medical Sciences, Guangzhou, Guangdong, China

**Keywords:** anomalous aortic origin of the right coronary artery (AAORCA), clinical decision-making, coronary artery anomalies, pediatric, real-world study, risk stratification

## Abstract

**Background:**

Anomalous aortic origin of the right coronary artery (AAORCA) is increasingly identified in children, yet risk stratification remains controversial. This study aims to identify key anatomical predictors of clinical symptoms and evaluate real-world management patterns in a large pediatric cohort.

**Methods:**

We conducted a single-center retrospective cohort study in pediatric patients ≤18 years diagnosed with AAORCA between November 2021 and December 2025. Clinical presentation, imaging findings, management strategies, and follow-up outcomes were analyzed. Integrated anatomical features were derived using a predefined hierarchical approach incorporating surgical findings, CTA, and echocardiography. Multivariable logistic regression analysis was performed to identify factors associated with symptomatic presentation.

**Results:**

A total of 151 patients were included (mean age 7.40 ± 4.87 years; 60.9% male), of whom 51.7% presented with symptoms. High-risk anatomical features were frequently identified but showed incomplete concordance with clinical symptoms. In multivariable analysis, patient age was independently associated with symptom presentation. Although ostial stenosis showed a stronger unadjusted association with symptoms than other anatomical features, no anatomical variable remained independently associated with symptom status after adjustment. A real-world decision pathway revealed substantial heterogeneity in management strategies among patients with high-risk anatomical findings. No sudden cardiac death or major adverse cardiovascular events were observed during available follow-up.

**Conclusion:**

Pediatric AAORCA demonstrates marked heterogeneity in clinical presentation, anatomical features, and management strategies. Clinical symptoms show incomplete concordance with high-risk anatomical features, highlighting the complexity of risk stratification in children. Among the evaluated clinical and anatomical characteristics, patient developmental age emerged as a strong independent predictor of symptoms, whereas ostial stenosis demonstrated the strongest unadjusted association with symptom status; however no anatomical variable remained independently associated with symptoms after multivariable adjustment. Real-world management strategies varied substantially and reflected individualized decision-making in the setting of limited pediatric evidence. Future studies may benefit from phenotype-oriented rather than anatomy-only risk classification frameworks.

## Introduction

Anomalous aortic origin of a coronary artery (AAOCA) is a relatively rare congenital coronary anomaly associated with a potential risk of life-threatening events, with a reported prevalence of approximately 0.17%–5.6% in the pediatric population (1). Among its subtypes, anomalous aortic origin of the right coronary artery (AAORCA) is among the most common (2). With the widespread use of advanced imaging modalities, including echocardiography and computed tomography angiography (CTA), an increasing number of pediatric cases are incidentally identified in asymptomatic individuals or those with nonspecific symptoms. While this has improved detection rates, it has also introduced new challenges in risk stratification and clinical management.

Although most pediatric patients with AAORCA have a favorable prognosis and the overall risk of sudden cardiac death (SCD) in asymptomatic individuals is considered low (3), the clinical presentation is highly heterogeneous. A subset of patients may develop exertional chest pain (4), syncope (5), or even SCD (6). This broad clinical spectrum, ranging from asymptomatic to life-threatening events, complicates risk assessment and limits the reliability of symptom-based stratification alone. Consequently, the management of asymptomatic or mildly symptomatic pediatric patients remains controversial, and current guidelines and studies have not reached consensus regarding surgical indications (1). Previous studies have suggested that the highest risk period occurs between 10 and 30 years of age (7), leading some investigators to even propose that younger children without high-risk clinical events may not require early surgical intervention (8).

Despite growing recognition of AAORCA, data remain limited, particularly in pediatric populations. Most existing studies have focused on macro-anatomical descriptions or surgical technicalities, leaving a substantial knowledge gap regarding the profound discordance frequently observed between clinical symptoms and gross anatomical classifications. Crucially, current management frameworks remain limited by an inability to determine whether traditional high-risk anatomical features, such as interarterial and intramural courses, adequately explain symptom presentation in children, or whether other anatomical characteristics may be more closely associated with clinical manifestations. This uncertainty contributes to ongoing controversy regarding risk stratification and treatment selection in pediatric AAORCA.

In this context, we conducted a retrospective cohort study of pediatric patients with AAORCA at our center. We aimed to systematically characterize clinical and imaging features, evaluate follow-up outcomes, and delineate real-world decision-making pathways. We further sought to explore the relationship between integrated anatomical findings and symptom status, with particular attention to the potential discordance between traditional anatomical risk features and clinical presentation. Our findings may provide clinically relevant evidence to support more individualized risk stratification and management strategies in pediatric AAORCA.

## Materials and methods

### Study design and patient selection

This was a single-center retrospective cohort study. Pediatric patients diagnosed with AAORCA at our center between November 2021 and December 2025 were identified through the electronic medical record system. Diagnosis was established by echocardiography and/or CTA. Patients who had been diagnosed prior to November 2021 and were under continued follow-up at our institution were also included.

Inclusion criteria were as follows: (1) age ≤ 18 years; (2) imaging-confirmed anomalous aortic origin of the right coronary artery from an abnormal anatomical location.

Exclusion criteria included: (1) presence of complex congenital heart disease (e.g., Double Outlet of the Right Ventricle, Tetralogy of Fallot, Pulmonary Atresia); and (2) insufficient imaging data to confirm the diagnosis. Patients with minor structural heart defects (e.g., atrial septal defect or ventricular septal defect) or a history of catheter ablation for arrhythmia were not excluded. The study was approved by the Ethics Committee of Guangdong Provincial People's Hospital (Approval No. KY2024-636-01).

### Clinical data collection

Demographic characteristics, initial clinical presentation (including chest pain, syncope, and exercise intolerance), and family history of sudden cardiac death (SCD) were retrospectively collected. For patients who underwent electrocardiography (ECG), the QTc interval, ST–T abnormalities, and arrhythmias were recorded. Sinus tachycardia was explicitly excluded from the classification of arrhythmias. This exclusion was based on the fact that an elevated heart rate (>100 bpm) frequently represents an age-specific normal physiological variant in younger pediatric populations or a transient reactive response (e.g., due to anxiety or crying during examination) rather than a primary clinical cardiac conduction anomaly. Cardiac biomarkers, including troponin and N-terminal pro-brain natriuretic peptide (NT-proBNP), were collected to assess myocrdial injury and cardiac function. An abnormal elevation of NT-proBNP was defined as >125.0 pg/mL, the institutional clinical cutoff. Pathological elevation of cardiac troponin T was defined as >14.0 pg/mL according to the reference range established by our central laboratory. These biochemical thresholds were applied uniformly across the entire study cohort.

Given the retrospective design, some variables were not routinely assessed or were incompletely documented, leading to missing data. Missing values were treated as unavailable and were primarily observed in outpatient settings.

### Imaging assessment

All patients underwent transthoracic echocardiography as the initial imaging modality to assess the coronary artery origin. A subset of patients further underwent cardiac or coronary CTA to obtain more detailed anatomical information. All patients who underwent surgical intervention had preoperative CTA, whereas CTA in non-surgical patients was performed selectively based on clinical indications.

CTA examinations were performed using either a Somatom Definition Flash scanner (Siemens Healthineers, Forchheim, Germany) or an Apex Expert CT scanner (GE Healthcare, USA). Both protocols employed prospective ECG-gating. Images were acquired with slice thicknesses of 0.6 mm (Siemens) or 0.625 mm (GE) and reconstructed using vendor-specific reconstruction algorithms (SAFIRE iterative reconstruction, strength level 3 with I26 kernel for Siemens; deep-learning image reconstruction, high-strength setting for GE). All CTA studies were independently reviewed by two cardiovascular radiologists. Because image interpretation was performed as part of routine clinical practice, reviewers had access to the imaging request forms and to the relevant clinical information available at the time of the examination. In cases of disagreement, a third senior cardiovascular imaging specialist adjudicated the findings, and the final anatomical classification was established by consensus.

Imaging assessments included the origin of the right coronary artery and high-risk anatomical features, such as an interarterial or intramural course and ostial stenosis. The interarterial course is defined as the anomalous coronary artery coursing within the space between the pulmonary artery and the ascending aorta. Intramural course is defined as the proximal portion of the anomalous coronary artery being embedded within the tunica media of the aortic wall, sharing a common wall with the aorta (6). For intramural course assessment, echocardiographic findings were prioritized when surgical confirmation was unavailable. This feature-specific hierarchy was predefined before data analysis. Due to variability in terminology across imaging reports, a unified definition was applied, and findings described as slit-like ostium, ostial narrowing, or reduced luminal diameter were categorized as ‘ostial stenosis.’

### Management strategy and follow-up

The decision to perform surgical intervention was determined through an interdisciplinary consensus-driven approach by a clinical team comprising senior attending pediatric cardiologists and pediatric cardiac surgeons, in close consultation with cardiovascular radiologists. Given that current international guidelines leave significant gray areas regarding surgical indications for certain pediatric AAORCA presentations, therapeutic decisions at our center were individualized and consensus-driven. The expert team synthesized available guideline frameworks with patient-specific risk factors, including clinical symptoms, advanced imaging findings, high-risk anatomical features, and parental preferences.

Anatomical risk classification was based on a hierarchical integration of available data sources, with intraoperative findings considered the reference when available, followed by CTA and echocardiography.

Patients were categorized into three groups according to management strategy: (1) surgical intervention; (2) surgery recommended but declined by guardians; and (3) conservative management. Follow-up data were obtained from outpatient visits and institutional medical records.

For patients who underwent surgery, surgical techniques and intraoperative anatomical findings were recorded. Consequently, surgical anatomical data were available only in the operated subgroup. Patients managed conservatively were advised to avoid strenuous exercise and undergo regular follow-up.

### Statistical analysis

Statistical analyses were performed using SPSS (version 20; IBM Corp, Armonk, NY). For the integrated anatomical analysis, a hierarchical data selection strategy was applied to ensure maximal diagnostic accuracy: surgical findings were prioritized as the gold standard, followed by CTA, and then echocardiography (except for intramural course, where echocardiography was prioritized over CTA).

Given the study's retrospective design, missing values were handled using an available-case analysis, in which the statistical analysis for each variable was performed using all available data points. No statistical imputation methods were applied to handle missing data. Consequently, the denominator varies across different analyzed variables depending on data availability, as explicitly noted in the respective table footnotes.

Continuous variables were assessed for normality using the Kolmogorov–Smirnov test. Normally distributed data are presented as mean ± standard deviation (SD) and compared using the independent-samples t-test; non-normally distributed data are expressed as median (interquartile range [IQR]) and compared using the Mann–Whitney U test. Categorical variables are presented as counts and percentages. Between-group comparisons of anatomical features and clinical characteristics were performed using the Pearson's Chi-square test or Fisher's exact test, as appropriate.

To identify factors associated with symptomatic presentation, univariable logistic regression analyses were first performed. Variables with clinical relevance were subsequently entered into a multivariable logistic regression model using the forced-entry (Enter) method, including age, sex, interarterial course, intramural course, and ostial stenosis. Results were reported as odds ratios (ORs) with 95% confidence intervals (CIs). A two-sided *P* value <0.05 was considered statistically significant.

## Results

### Patient characteristics

A total of 151 pediatric patients with AAORCA were included in this study, comprising 92 males (60.9%) and 59 females (39.1%), with a mean age at diagnosis of 7.40 ± 4.87 years. Overall, 78 patients (51.7%) presented with clinical symptoms, with chest pain being the most common manifestation; the remaining 73 patients (48.3%) were asymptomatic and were mostly identified incidentally during routine examinations or evaluation for unrelated conditions. No patients had a documented family history of SCD.

In terms of laboratory and electrocardiographic evaluation, a subset of patients demonstrated evidence of myocardial injury or electrophysiological abnormalities. Among the 87 patients who underwent NT-proBNP testing, 22 (25.3%) showed elevated levels. Notably, when stratified by clinical presentation, the rate of NT-proBNP elevation was significantly higher in the asymptomatic group than in the symptomatic group [53.3% (16/30) vs. 10.5% (6/57), *P* < 0.001]. Similarly, among the 73 patients tested for cardiac troponin T, 12 (16.4%) exhibited elevated levels. A similar and unexpected disparity was observed between the two subgroups, with the tested asymptomatic patients demonstrating a significantly higher rate of troponin T elevation compared to their symptomatic counterparts [33.3% (9/27) vs. 6.5% (3/46), *P* = 0.0006]. The distinct testing denominators and detailed distribution of these biomarkers are comprehensively summarized in Table 1. Among the 131 patients with available electrocardiographic data, QTc prolongation was observed in 9 patients (7.0%), ST–T abnormalities in 23 (17.7%), Q waves in 3 (2.3%), and arrhythmias in 36 (27.5%). Arrhythmia types included right bundle branch block, atrioventricular block, and premature beats, with 1 case of ventricular tachycardia. In addition, echocardiographic functional assessment revealed that a minority of patients had impaired cardiac function; reduced LVEF was observed in 6 patients (4.0%) (Table 1).

**Table 1:** Baseline clinical characteristics and echocardiographic features in pediatric patients with AAORCA.

Characteristics	Overall (*N* = 151)	Symptomatic (*N* = 78)	Asymptomatic (*N* = 73)	*P* value
Demographics				
Male, n (%)	92 (60.9%)	52 (66.7%)	40 (54.8%)	0.135
Age at diagnosis (years)	7.40 ± 4.87	10.63 ± 3.49	3.93 ± 3.59	＜0.001
Clinical Presentation				
Symptomatic, n (%)	78 (51.7%)	78 (100.0%)	0	＜0.001
Chest pain	43/78 (55.1%)	43 (55.1%)	0	
Syncope	8/78 (10.3%)	8 (10.3%)	0	
Exercise intolerance	10/78 (12.8%)	10 (12.8%)	0	
Asymptomatic, n (%)	73 (48.3%)	0	73 (100.0%)	＜0.001
Cardiac Biomarkers				
Elevated Troponin Level	12/73 (16.4%)	3/46 (6.5%)	9/27 (33.3%)	0.006
Elevated NT-proBNP	22/87 (25.3%)	6/57 (10.5%)	16/30 (53.3%)	＜0.001
Electrocardiography				
QTc,ms (*n* = 127)	406.31 ± 35.43	407.06 ± 29.31	405.41 ± 41.82	0.796
QTc prolongation	9/128 (7.0%)	4/70 (5.7%)	5/58 (8.6%)	0.731
ST-T abnormality	23/130 (17.7%)	15/72 (20.8%)	8/58 (13.8%)	0.296
Q wave	3/131 (2.3%)	2/73 (2.7%)	1/58 (1.7%)	1.000
Arrhythmia	36/131 (27.5%)	25/73 (34.2%)	11/58 (19.0%)	0.052
Echocardiographic functional assessment				
LVEF, %	69.10 ± 8.96	68.88 ± 10.67	69.33 ± 6.69	0.755
LVEF decreased, n (%)	6 (4.0%)	4 (5.3%)	2 (2.7%)	0.682

Data are presented as mean ± standard deviation or n (%). The denominator for each variable reflects the number of patients with available data due to the retrospective nature of the study. NT-proBNP, N-terminal pro-brain natriuretic peptide; LVEF, left ventricular ejection fraction.

Statistical analysis revealed significant demographic and biochemical differences between the clinical subgroups. Patients in the symptomatic group were significantly older than those in the asymptomatic group at presentation (*P* < 0.01). A counterintuitive trend was observed in cardiac biomarkers: the asymptomatic group had significantly higher rates of elevated Troponin T and NT-proBNP than the symptomatic group. Furthermore, although the prevalence of arrhythmias was higher in symptomatic patients (34.2% vs. 19.0%), this difference was only marginally significant (*P* = 0.052).

### Imaging characteristics

**Figure 1:**
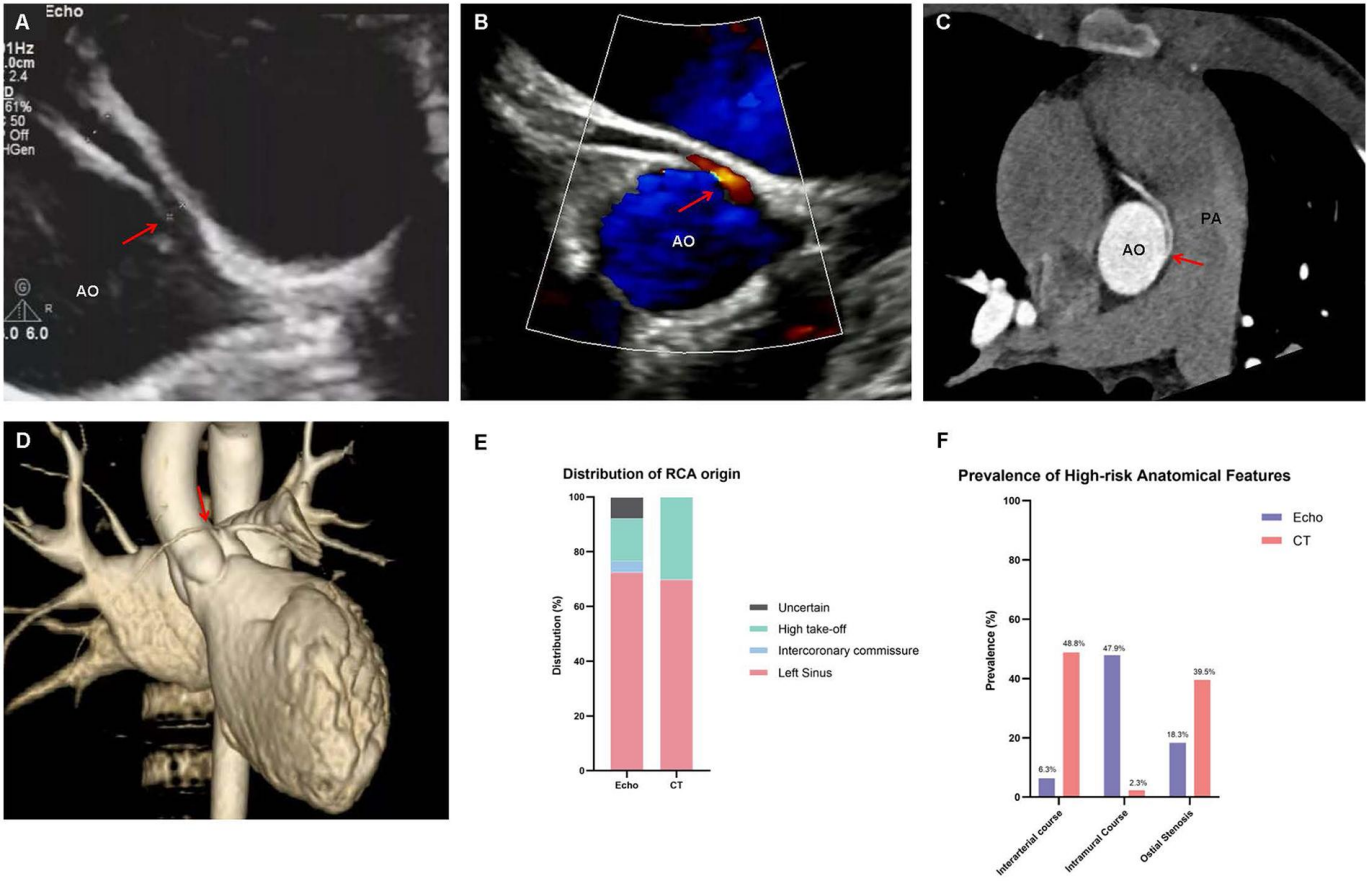
Representative imaging findings and distribution of anatomical features in pediatric patients with AAORCA. **(A)** Transthoracic echocardiographic image demonstrating an intramural course of the right coronary artery (RCA) with proximal ostial narrowing (red arrow). **(B)** Color Doppler echocardiography demonstrating accelerated flow at the proximal intramural RCA segment (red arrow), indicating ostial stenosis. **(C)** CT image showing a high take-off RCA with an interarterial course between the aorta and pulmonary artery (red arrow). **(D)** CT reconstruction demonstrating anomalous origin of the RCA from the left coronary sinus with ostial stenosis (red arrow). **(E)** Distribution of RCA origin assessed by echocardiography and CT. **(F)** Prevalence of imaging-derived anatomical features associated with potential clinical risk, including interarterial course, intramural course, and ostial stenosis, across imaging modalities. LCA, left coronary artery; AO, aorta; PA, pulmonary artery; Echo, echocardiography; CT, computed tomography; RCA, right coronary artery.

Echocardiography suggested or confirmed AAORCA in 142 patients (94.0%). The most common origin was the left coronary sinus (72.5%), followed by a high-take-off origin (15.5%). In 7.7% of cases, although AAORCA was suspected, the exact origin could not be determined (Figure 1). Regarding high-risk anatomical features, echocardiography identified an intramural course in approximately half of patients, whereas the detection rates for interarterial course and ostial stenosis were relatively low.

A total of 129 patients (85.4%) underwent cardiac or coronary CTA, all of whom had clearly defined coronary origin, indicating improved anatomical delineation compared with echocardiography. CTA demonstrated that the right coronary artery most commonly originated from the left coronary sinus or presented as a high take-off origin. Regarding high-risk anatomical features, an interarterial course was observed in 48.8% of patients and ostial stenosis in 39.5%, whereas an intramural course was rarely identified (Figure 1).

### Real-world management strategies

**Figure 2:**
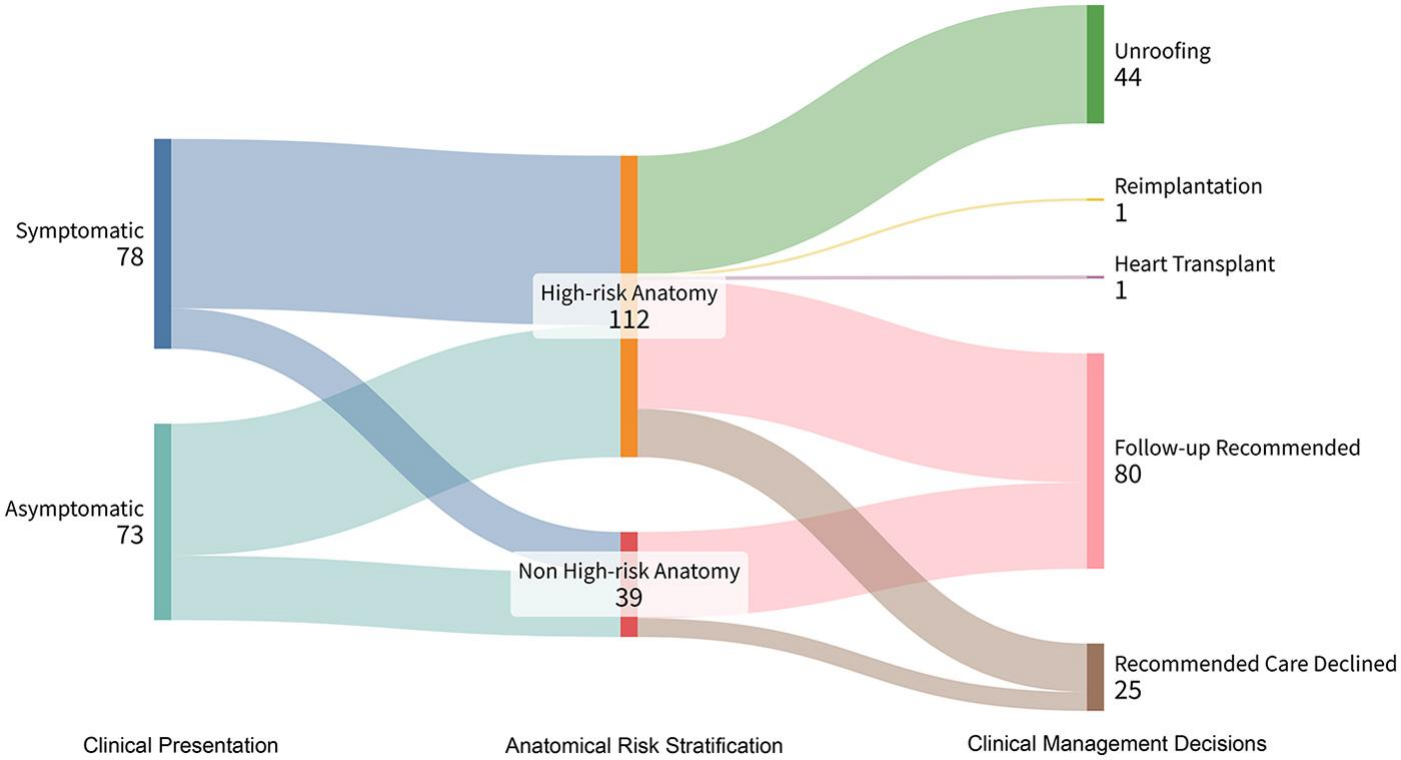
Real-world clinical decision pathways in pediatric patients with AAORCA. Sankey diagram illustrating patient flow from clinical presentation to anatomical risk stratification and subsequent management strategies. Flows from left to right map the progression from initial clinical presentation (78 symptomatic vs. 73 asymptomatic status) through multi-modality imaging phenotypes (112 with high-risk anatomy vs. 39 non-high-risk anatomy), culminating in final therapeutic management strategies (surgical repair in 46 cases including 44 unroofing, 1 reimplantation and 1 heart transplant vs. conservative surveillance in 105 including 25 declined recommended care and 80 under follow-up surveillance). Flow widths are proportional to patient numbers.

Among the 151 patients, 63 (41.7%) were symptomatic and had high-risk anatomical features, 49 (32.5%) were asymptomatic but had high-risk features, and 15 (9.9%) were symptomatic without high-risk anatomical findings. A total of 46 patients (30.5%) underwent surgical intervention, all of whom had high-risk anatomical features. The predominant surgical technique was unroofing (95.7%) (Table 2). Notably, 25 non-surgical patients were recommended for further evaluation or surgical intervention, but their parents declined these recommendations. In addition, a considerable proportion of patients with high-risk anatomical features were managed conservatively (Figure 2).

**Table 2:** Surgical procedure and intraoperative findings.

Characteristics	Surgical Group (*N* = 46)
Surgical Technique	
Unroofing	44 (95.7%)
Reimplantation	1 (2.2%)
Heart Transplantation	1 (2.2%)
Origin (*n* = 45^a^)	
Left sinus	39/45 (86.7%)
Intercoronary commissure	1/45 (2.2%)
High take-off	5/45 (11.1%)
High-risk Anatomy (*n* = 45^a^)	
Interarterial Course	13/45 (28.9%)
Intramural Course	44/45 (97.8%)
Ostial Stenosis	40/45 (88.9%)

a One patient who underwent heart transplantation was unavailable for intraoperative coronary origin and course.

### Integrated anatomical features comparison between symptomatic and asymptomatic groups

To evaluate the clinical significance of coronary anomalies, we analyzed integrated anatomical features derived from a multimodality approach, prioritizing surgical findings over CTA and echocardiography as defined in the Methods.

**Figure 3:**
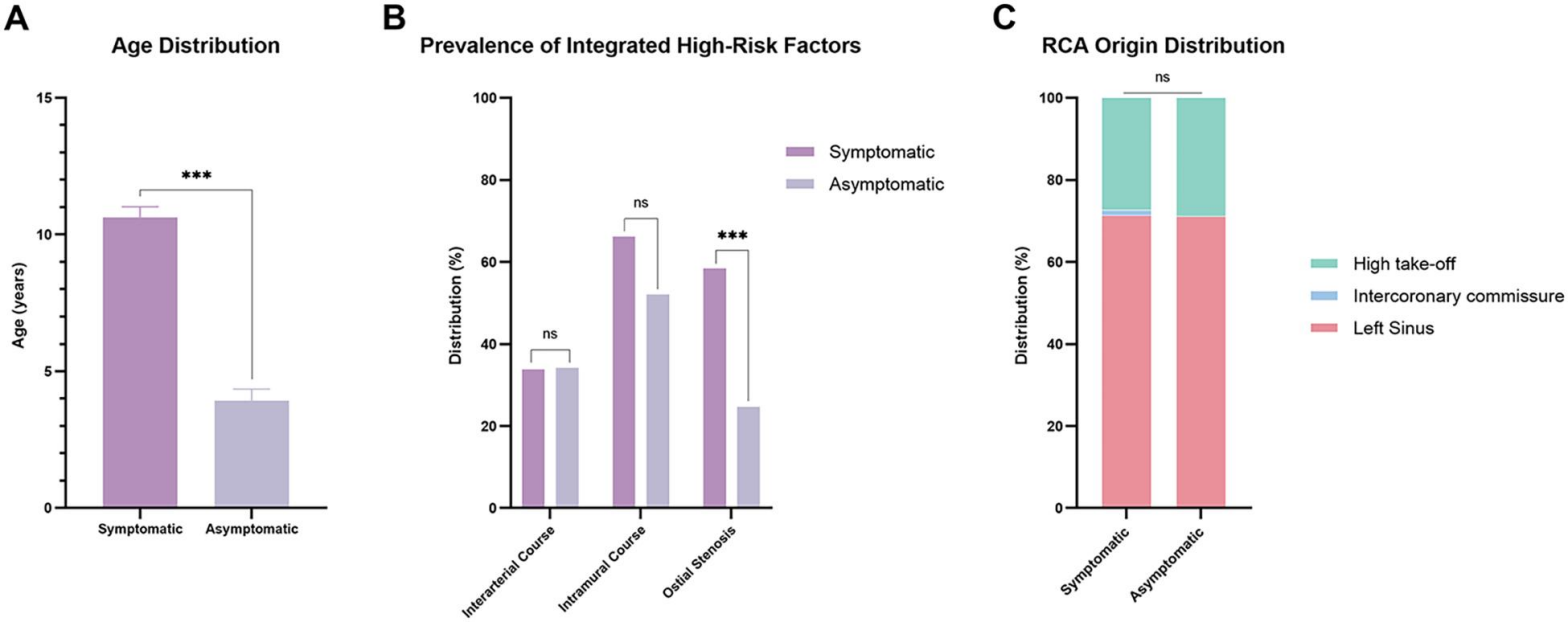
Comparison of demographic and integrated anatomical features between symptomatic and asymptomatic patients. Integrated anatomical features were determined using a predefined hierarchical diagnostic strategy. In patients who underwent surgery, intraoperative findings were considered the reference standard. For non-surgical patients, CT findings were used when available. Echocardiographic findings were used only when CT was unavailable. For intramural course assessment, echocardiographic findings were prioritized over CT because intramural segments were infrequently identified by CT in our cohort. **(A)** Mean age comparison between groups. **(B)** Prevalence of integrated anatomical high-risk features. **(C)** Distribution of coronary artery origins across the two groups. Data are presented as Mean ± SEM. NS, not significant; ***, *P* < 0.001.

Symptomatic patients were significantly older than asymptomatic patients at presentation. In univariable analysis, ostial stenosis was associated with a higher prevalence in symptomatic patients compared with asymptomatic patients (*P* < 0.001). However, other traditional high-risk anatomical features, including the interarterial and intramural courses, showed no significant association with symptom status. Furthermore, positional anomalies (Left sinus, Commissure, or High take-off) showed no significant correlation with clinical symptoms (Figure 3).

To identify factors associated with symptom presentation while accounting for potential confounders, a multivariable binary logistic regression model was constructed (Table 3). Patient age was independently associated with symptom presentation (OR = 1.42, 95% CI: 1.26–1.60, *P* < 0.001). Ostial stenosis showed the strongest association among the evaluated anatomical variables; however, this association was attenuated after multivariable adjustment and did not reach statistical significance (OR = 0.40, 95% CI: 0.15–1.01, *P* = 0.052). Consistent with the univariable analysis, neither interarterial course (*P* = 0.938) nor intramural course (*P* = 0.496) was independently associated with symptom presentation.

**Table 3:** Univariable and multivariable logistic regression analysis for symptomatic presentation.

Variable	Univariable OR (95% CI)	*P* Value	Multivariable OR (95% CI)	*P* Value
Age (years)	1.44 (1.29–1.62)	<0.001	1.42 (1.26–1.60)	<0.001
Male sex	1.65 (0.85–3.19)	0.136	0.93 (0.39–2.25)	0.874
Interarterial course	0.98 (0.50–1.92)	0.951	1.04 (0.42–2.59)	0.938
Intramural course	1.81 (0.94–3.49)	0.078	0.73 (0.29–1.83)	0.496
Ostial stenosis	4.30 (2.14–8.65)	<0.001	0.40 (0.15–1.01)	0.052

### Follow-up outcomes

No perioperative mortality was observed. During follow-up, no cases of SCD or major adverse cardiovascular events were reported.

Among the 46 surgically treated patients, one patient continued to experience recurrent syncope postoperatively and was subsequently found to carry a pathogenic KCNQ1 mutation; this patient is currently receiving *β*-blocker therapy. One patient experienced transient chest pain after surgery, and another reported three episodes of syncope during follow-up, although no further intervention was pursued.

Among the 105 nonsurgical patients, overall clinical status remained stable. Only two patients developed new-onset chest pain, both of whom declined surgical intervention. All non-surgical patients were advised to avoid strenuous physical activity and to undergo regular follow-up.

## Discussion

In this real-world cohort of pediatric AAORCA, we demonstrate that clinical symptoms, anatomical risk features, and management decisions are frequently decoupled rather than aligned, challenging the conventional paradigm that these domains follow a linear and hierarchical relationship. Instead, our findings suggest that risk assessment and clinical decision-making in pediatric AAORCA are inherently multidimensional and cannot be adequately captured by any single axis of evaluation.

Although AAORCA has traditionally been considered a lower-risk entity compared with anomalous origin of the left coronary artery, the presence of syncope and SCD in selected cases continues to underscore the importance of accurate risk stratification (9, 10). In this context, our study demonstrates that clinical symptoms and high-risk anatomical features frequently do not align, representing a key challenge in pediatric practice. The pronounced ‘age gap' between our symptomatic and asymptomatic cohorts—averaging 10.6 and 3.9 years, respectively—provides critical insight into the natural history of pediatric AAORCA. The delayed onset of symptoms might be attributed to increased physical demands that exacerbate coronary ostial compression. Alternatively, this gap may reflect cognitive and communicative limitations in early childhood, where younger patients are unable to articulate or localize ischemic distress. This suggests that a 'silent' period in early childhood does not preclude future risk, necessitating longitudinal surveillance as patients enter more active developmental stages. Furthermore, the discrepancy observed between echocardiography and CTA in assessing the intramural course underscores the importance of integrating multimodality imaging to minimize modality-dependent diagnostic variability.

Specifically, we observed that some patients with high-risk anatomical features remained asymptomatic, while others with symptoms did not exhibit typical high-risk imaging findings. This discordance between symptoms and anatomy suggests that reliance on either dimension alone may lead to misclassification of risk. We also observed higher rates of Troponin T and NT-proBNP elevation among the tested asymptomatic patients. However, this finding should be interpreted with considerable caution. Because the study was retrospective, biomarker testing was not performed systematically and was left to the clinician's discretion rather than a standardized protocol. Consequently, only a subset of patients underwent testing, introducing potential selection bias. The asymptomatic patients who underwent biomarker assessment may therefore not be representative of the overall asymptomatic cohort. As a result, these findings should not be interpreted as evidence of widespread subclinical myocardial ischemia among asymptomatic children with AAORCA. Nevertheless, the observation that biomarker abnormalities were identified in some asymptomatic patients highlights the limitations of relying solely on symptoms for risk assessment. This concept is consistent with previous reports showing that conventional tools such as ECG and exercise stress testing may fail to identify a proportion of patients at risk for myocardial ischemia or sudden cardiac death (11). Taken together, these findings support a more comprehensive risk assessment framework that integrates clinical presentation, anatomical characteristics, and functional evaluation, including hemodynamic assessment (12, 13), rather than relying on any single indicator.

Traditional risk stratification for AAORCA has leaned heavily on anatomical descriptions; however, our findings suggest that the relationship between coronary anatomy and symptom presentation may be more complex than previously appreciated. We observed a notable dissociation between traditional high-risk anatomy and clinical symptoms, with neither interarterial course nor intramural course independently associated with symptomatic presentation in the multivariable model. In contrast, ostial stenosis demonstrated the strongest association with symptom status in univariable analysis, but this association was attenuated after adjustment for age, sex, and other anatomical variables (*P* = 0.052). Current guidelines and expert consensus documents (14–16) continue to regard interarterial course, intramural course, and ostial abnormalities as important anatomical considerations in risk stratification; however, these criteria were largely derived from broader AAOCA populations and subsequently extrapolated to AAORCA. In keeping with previous pediatric AAORCA cohorts that reported heterogeneous clinical presentations and inconsistent associations between anatomical features and symptoms (17, 18), our findings further support the notion that individual anatomical markers alone may have limited ability to discriminate symptomatic from asymptomatic patients. Our results do not suggest that interarterial or intramural courses lack clinical relevance; rather, they indicate that these features may be insufficient as standalone determinants of symptom expression in pediatric AAORCA. Similarly, although ostial stenosis showed the strongest association with symptoms in our cohort, the attenuation of statistical significance after multivariable adjustment suggests that this relationship is likely influenced by clinical covariates, particularly age, and should therefore be interpreted cautiously.

Beyond risk assessment, our study provides novel insight into real-world decision-making behavior. By reconstructing clinical pathways using a Sankey-based model, we show that patients with comparable anatomical risk profiles frequently undergo divergent management strategies, including surgical intervention, conservative follow-up, or refusal of recommended treatment. This 'similar anatomy, different decisions' pattern reflects not only variability in clinical judgment but also the inherent limitations of current guideline-based stratification in pediatric populations. Although most non-surgical patients in our cohort remained clinically stable and no cases of sudden cardiac death or major adverse cardiovascular events were observed during follow-up, this indicates a relatively low short-term event rate in children compared with adults (19). This must be interpreted strictly as a localized, short-term observational finding rather than a definitive indication of long-term safety. This low event rate within our study window should not, under any circumstances, be interpreted as an absence of catastrophic risk or used to provide false clinical reassurance. The relatively short follow-up duration, combined with the low incidence of adverse events, limits the ability to fully assess long-term outcomes. Consequently, clinical surveillance must remain vigilant, as a benign pediatric course does not preclude dynamic changes in geometry or subsequent risk expansion. Moreover, the potential impact of prolonged exercise restriction and delayed intervention on psychosocial development and cardiovascular health warrants further consideration (20, 21).

Regarding our primary surgical findings, our data highlight that operative intervention does not uniformly or immediately guarantee complete symptom resolution. Among the 46 patients who underwent surgical repair in this cohort, post-procedural clinical manifestations were meticulously documented, including one case of transient postoperative chest pain and two patients who experienced episodes of syncope. Most notably, one patient continued to suffer from recurrent syncope after technically successful surgical unroofing and was subsequently identified as carrying a pathogenic KCNQ1 ion-channel mutation.

When benchmarking these internal outcomes against the existing literature, our observed postoperative event rate appears markedly lower than previously reported. Previous studies have reported persistent or newly developed symptoms after surgery (17, 22), and in some reports, persistent or recurrent symptoms have been observed in more than 25% of patients after surgery (23). This stark contrast between our low complication rate and the high rates reported in the literature underscores the profoundly multifactorial and heterogeneous nature of pediatric symptom generation (24, 25). Our unique discovery of the KCNQ1 mutation in a persistently symptomatic patient provides concrete evidence that physicians must avoid the clinical trap of attributing all ischemic or syncopal distress solely to coronary structural anomalies, especially when the presentation is atypical or fails to resolve following appropriate surgical procedures. Comprehensive electrophysiological and alternative workups are strongly warranted in these discordant cases.

## Study limitations

This study has several limitations. As a retrospective single-center analysis, it is subject to potential selection bias. The availability of biomarker testing and imaging examinations was not standardized across the cohort, which may have introduced selection and measurement bias and affected subgroup comparisons. To reduce variability in anatomical characterization, we used a predefined hierarchical integration strategy prioritizing intraoperative findings, followed by CTA and echocardiography. Finally, the relatively short follow-up duration limits assessment of long-term outcomes. Future multicenter prospective studies with standardized diagnostic protocols and extended follow-up are warranted.

## Conclusion

This study demonstrates that pediatric AAORCA is characterized by marked clinical and anatomical heterogeneity. Our findings highlight an incomplete concordance between traditional high-risk anatomical features and clinical presentation. Patient developmental age emerged as a strong independent predictor of symptoms, whereas ostial stenosis showed the strongest association with symptom status among the evaluated anatomical features, although this association was attenuated after multivariable adjustment. While short-term outcomes remained favorable during follow-up under real-world management, our findings support a refined, individualized assessment strategy that emphasizes detailed evaluation of coronary morphology, particularly the coronary orifice, alongside clinical characteristics. Future studies may benefit from phenotype-oriented rather than anatomy-only risk stratification frameworks.

## Data Availability

The original contributions presented in the study are included in the article/Supplementary Material, further inquiries can be directed to the corresponding authors.
